# Axillary intra-aortic balloon pump, biventricular assist device implantation and subsequent orthotopic heart transplantation in a patient with sickle cell trait

**DOI:** 10.1093/jscr/rjac260

**Published:** 2022-06-18

**Authors:** Marcus Taylor, Zakariya Mouyer, Paul Callan, Steve Shaw, Rajamiyer Venkateswaran, Nnamdi Nwaejike

**Affiliations:** Department of Cardiothoracic Transplantation, Manchester University Hospital NHS Foundation Trust, Wythenshawe Hospital, Manchester, UK; Department of Cardiothoracic Transplantation, Manchester University Hospital NHS Foundation Trust, Wythenshawe Hospital, Manchester, UK; Department of Cardiothoracic Transplantation, Manchester University Hospital NHS Foundation Trust, Wythenshawe Hospital, Manchester, UK; Department of Cardiothoracic Transplantation, Manchester University Hospital NHS Foundation Trust, Wythenshawe Hospital, Manchester, UK; Department of Cardiothoracic Transplantation, Manchester University Hospital NHS Foundation Trust, Wythenshawe Hospital, Manchester, UK; Department of Cardiothoracic Transplantation, Manchester University Hospital NHS Foundation Trust, Wythenshawe Hospital, Manchester, UK

**Keywords:** heart transplantation, mechanical circulatory support, axillary intra-aortic balloon pump, sickle cell disease

## Abstract

A 38-year-old male with sickle cell trait and acute refractory heart failure received an axillary intra-aortic balloon pump and short-term biventricular assist device. He underwent orthotopic heart transplantation 45 days later, which was complicated by major bleeding necessitating significant intra-operative transfusion. Support with veno-arterial extracorporeal membrane oxygenation was provided and successfully weaned five days later. He made a full recovery and remains alive and well 34 months after discharge. We hypothesize that the protective peri-operative measures undertaken, including normothermia during surgery and post-operative haemodynamic stability due to the use of mechanical circulatory support, conveyed a degree of protection against complications associated with sickle cell dysfunction and contributed to the successful outcome.

## INTRODUCTION

Sickle cell disease is an autosomal recessive disorder characterized by a replacement of the normal HbA protein with a mutated HbS protein. The subsequent disruption to red cell architecture can cause anaemia and thrombosis [1]. Patients classified as sickle cell trait (SCT) carriers are frequently asymptomatic, with the limited presence (often 30–45% of total) of the HbS gene [2] often the only abnormality identified.

Patients with sickle cell dysfunction (SCD) are recognized as high risk for cardiac surgery, as many of the factors which precipitate complications associated with SCD are routinely encountered during cardiopulmonary bypass (CPB), including hypothermia, stress, inflammation and acidosis [3]. Whilst formal guidance is lacking, anecdotal evidence suggests that many UK centres perform pre-operative exchange transfusion in order to achieve an HbS percentage of less than 30% [4].

## CASE REPORT

**Figure 1:**
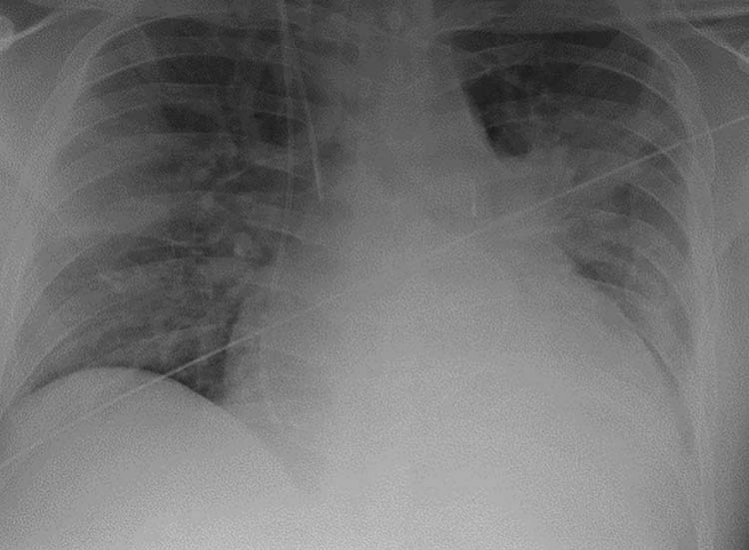
Chest radiograph demonstrating pulmonary oedema prior to implantation of biventricular assist device.

A 38-year-old Caucasian male presented to hospital with shortness of breath. He was diagnosed with dilated cardiomyopathy following transthoracic echocardiography. He continued to deteriorate and developed florid pulmonary oedema as demonstrated in Fig. 1, necessitating his transfer to our centre for further management. Prior to this presentation he had no known comorbidities.

**Figure 2:**
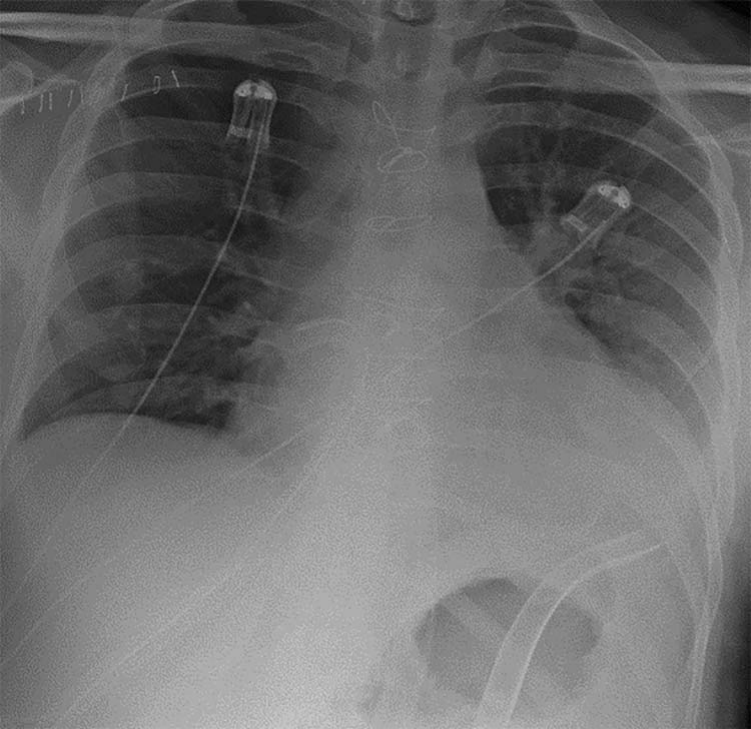
Chest radiograph demonstrating biventricular assist device pipes in situ and resolution of previous pulmonary oedema.

As part of his work-up for cardiac transplantation he was found to be a carrier of the HbS sickle cell trait, with an estimated HbS concentration of 34.4%. Due to his deteriorating condition and the uncertainty as to his candidacy for transplantation [5], the management plan was for mechanical circulatory support as a bridge-to-decision. An axillary IABP was inserted as previously described [6]. Unfortunately**,** he continued to deteriorate, requiring the implantation of a short-term BiVAD, as previously described [7]. His overall condition improved with BiVAD support—there was recovery of renal and liver function and resolution of pulmonary oedema, as shown in Fig. 2. However, there was no evidence of myocardial recovery, and he was therefore placed on the super-urgent heart transplant waiting list.

After 45 days receiving BiVAD support, he underwent orthotopic heart transplantation (HTX). The donor heart was from a 31-year-old male and was transported on cold storage. At the time of surgery his haemoglobin was 119 g/L, platelet count was 135 × 10 [9]/L, haematocrit was 0.357 L/L and mean cell volume was 79 fl. The patient remained normothermic (36.5°C) throughout the operation and diastolic arrest was initially achieved with cold blood cardioplegia delivered via the aortic root. Upon completion of all anastomoses, significant bleeding from the posterior aortic wall was encountered, which required a further period of aortic cross-clamping, cold blood cardioplegia and the administration of multiple blood products as part of the major haemorrhage protocol. Due to right ventricular dysfunction, the decision was taken to initiate central veno-arterial extracorporeal membrane oxygenation (VA-ECMO), which was performed uneventfully. VA-ECMO was weaned on the fifth post-operative day, and the patient was discharged from the critical care unit six days later. He was discharged home 28 days after HTX. Since discharge, he has continued to recover and remains alive and well 34 months after discharge.

## DISCUSSION

Reported series of patients undergoing cardiac surgery with concomitant SCD are not common and hence, universal agreement on the preferred protective strategy is lacking [5]. Compared to patients with sickle cell disease, patients with SCT are significantly less likely to experience true sickle cell crisis. Nevertheless, they have an increased risk of complications, particularly vascular and renal issues. Consequently, avoidance of the recognized factors associated with sickle cell crisis seems a prudent approach and is frequently advocated in studies published in this area of cardiac surgical practice [8]. Given that some contemporary cardiac surgery series have reported acceptable outcomes whilst maintaining normothermia [9], and that hypothermia causes vasoconstriction, which can precipitate sickle cell crisis, we chose to perform all cardiac surgical procedures on this patient with systemic normothermia.

Minimizing right ventricular dysfunction after HTX is usually managed with inhaled nitric oxide and intravenous phosphodiesterase inhibitors (PDI), such as milrinone [10]. Unfortunately, the use of PDIs is linked with higher rates of vaso-occlusive crisis [8]. Our patient experienced right ventricular dysfunction, necessitating VA-ECMO. In our centre we have a low threshold for employing post-operative VA-ECMO for HTX patients. Prompt decision-making to initiate peri-operative ECMO decreases our reliance on PDIs and may have contributed to our ability to successfully avoid sickle cell crisis in this patient.

Exchange transfusion is another strategy employed to reduce the risk of sickle cell crisis, although no randomized trials underpin this practice. Nevertheless, guidelines advocate the use of exchange transfusion to achieve an HbS level < 30% in high-risk situations, which includes cardiac surgery [11]. This is based on evidence linking higher concentrations of HbS with a greater chance of crisis and a greater pathological burden [3]. Alternative studies, not limited to cardiac surgery, instead suggest that transfusing to a Hb level > 10 g/L is associated with similar outcomes to those with HbS <30% [12]. Transfusion is not without risk, and given that there is evidence to suggest that adequate outcomes can be achieved without transfusion when homeostasis is appropriately maintained [13], current advice is to consider transfusion on an individual case-by-case basis. In this case, we transfused to a pre-operative Hb level of 119 g/L and did not perform formal exchange transfusion.

Cardiac transplantation in patients with sickle cell disease is rarely reported. Among the few published cases [5, 14, 15], strategies to avoid sickle cell crisis are varied and lack high-quality supporting evidence. We have described the first published case of a patient with SCT undergoing axillary IABP, BiVAD implantation and subsequent HTX. Despite a number of risk factors in this case associated with complications in patients with SCD, including intra-operative haemodynamic instability, use of PDIs and a pre-operative HbS fraction in excess of 30%, our patient remains alive and well almost three years after surgery. This case demonstrates that with appropriate assessment and careful intra-operative management, successful outcomes can be achieved, and sickle cell crisis avoided.
